# Normality data of eGFR and validity of commonly used screening tests for CKD in an area with endemic CKD of unknown etiology; need for age and sex based precise cutoff values

**DOI:** 10.1186/s12882-019-1477-9

**Published:** 2019-08-05

**Authors:** Nalaka Herath, Rajeewa Dassanayake, Manjula Dissanayake, Chamara Janitha, Kosala Weerakoon, Chalaka Kumarasinghe, Terence Gamini de Silva, Suneth Agampodi

**Affiliations:** 1 Teaching Hospital, Anuradhapura, Sri Lanka; 2 Teaching Hospital, Kandy, Sri Lanka; 3 Department of Parasitology, Faculty of Medicine and Allied Sciences, Rajarata University of Sri Lanka, Saliyapura, Sri Lanka; 4 Department of Community Medicine, Faculty of Medicine and Allied Sciences, Rajarata University of Sri Lanka, Saliyapura, Sri Lanka

**Keywords:** CKD, CKDu, eGFR, Sri Lanka, Anuradhapura, Baseline values, Screening, UACR, UPCR

## Abstract

**Background:**

Chronic Kidney Disease in certain part of Sri Lanka and increasing burden of CKD in some other countries is a global public health problem. While the underlying causes of majority of cases are unknown, effective control and prevention strategies are yet to be taken. Though the disease has been identify more than decade ago, baseline data on renal function are not available. This study reports the age and sex disaggregated data of renal functions among screening participants of the Anuradhapura, the district with the highest disease burden in Sri Lanka.

**Methods:**

The screening prorgramme was done as a part of CKD control programme of Anuradhapura. All screening centers were visited and information and urine sample collection tubes were distributed before the screening date. A serum and urine sample was taken from all participants. In a subsample, urine sulfosalicylic acid test (SSA Test), urine dipstick test, urine albumin to creatinine ratio (UACR) and urine protein to creatinine ratio (UPCR) was done.

**Results:**

The study sample included 7768 apparently healthy people aging 18 to 93 years and females (*n* = 5522) accounted for 71.1% of the sample. Mean age of the participants was 45.9 (SD 14.1) years. Mean eGFR in this population was 90.8 mL/min/1.73m^2^(SD 24.6) with a significantly lower eGFR (88.1 mL/min/1.73m^2^) among males compared to female (92.8 mL/min/1.73m^2^). Mean eGFR was 115 mL/min/1.73m^2^ (SE .5) among participants aging less than 30 and this value drastically reduced to 59.1 mL/min/1.73m^2^ (SE 1.2) among people aging more than 70 years. Proportion of people having reduction of eGFR compatible with mild, moderate, severe and kidney failure categories was 33.9(32.7–34.8), 8.4(7.8–9.0), 1.5(1.2–1.7) and 0.7(0.5–0.9). The age and sex adjusted prevalence of eGFR less than 60 mL/min/1.73 m2 in a single sample in this population was 10.6%. Bayesian Latent Class model analysis shows that UPCR> 150 has the highest sensitivity to detect those who are with eGFR less than 60 mL/min/1.73 m2. UACR, the usual recommended test as a screening test was having a sensitivity of 35.3% in this population.

**Conclusion:**

UPCR and UACR should be use as a screening tests in areas with high proportion of CKDu patients. More research are required to investigate the use of age and sex specific cut off values to diagnose CKD.

## Background

Chronic kidney disease (CKD) has become a major public health problem worldwide [1]. Epidemiological studies of the adult population in several countries report varying degree of CKD prevalence with 9–11% in United States (US) [2], 9.1% in Spain [3], and 10.6% in Netherlands [4]. CKD prevalence increases with age, and is highest after the age of 60 years [2–4]. There a regional variation in CKD is reported in several studies, which is attributed to socio demographics risk factors [5].

From the 1990s, an increase in CKD prevalence [2, 3, 6–8] and emergence of a CKD of unknown etiology (CKDu) was observed in several countries including El Salvador, Nicaragua, Costa Rica, Sri Lanka [9–11], Egypt [12] and India [13]. CKDu is usually asymptomatic until advanced disease. Clinically, CKDu is characterized by minimal or no urinary abnormalities due to chronic interstitial nephropathy, which has been confirmed by renal biopsies in Costa Rica, El Salvador and Sri Lanka [11].

The prevalence and associated mortality of CKD has been on the rise in Sri Lanka [8]. Exact prevalence of CKD in Sri Lanka is largely unknown, mainly due to the unavailability of renal registries and lack of epidemiological studies. However, population screening in Sri Lanka shows variable prevalence in different districts [8]. The prevalence of CKDu was 15.1% in Anuradhapura, 20.6% in Polonnaruwa and 22.9% in Badulla [8]. While the screening tests are in process, baseline data on the population estimated glomerular filtration rate (eGFR) distribution and age, sex disaggregated data on eGFR for Sri Lanka is not available in published literature. For clinical practice as well as for public policy, assessment of baseline eGFR is recommended. Planning of screening programmes, determining age and sex specific cut off points as well as public health programme planning should ideally be based on these baseline data.

Screening of high risk people for CKD has been a standard practice worldwide [14–16]. There is no uniform screening method to be applied to detect CKD patients with all different aetiologies. Urine protein analysis or serum creatinine estimation or combination of these two has been used to detect CKD in diabetes and hypertensive patients [17]. Commonly used screening tests for abnormal protein excretion in urine includes urine sulfosalicylic acid test (SSA Test), urine dipstick test, urine albumin to creatinine ratio (UACR) and urine protein to creatinine ratio (UPCR). Urine SSA tests and dipstick analysis is used in most outpatient settings for semi quantitative measurement of urine protein concentration. UPCR and UACR measure protein excretion quantitatively and can be done on a spot urine sample. An elevated UACR (or albuminuria) is the most widely used marker for identifying kidney damage, as it is highly sensitive in the earlier stages of traditional CKD [4]. Persistent microalbuninuria has been used to detect early CKD in diabetes and hypertensive patients. However, urine protein or microalbumin analysis may not be effective in screening of patients with CKDu.

Our study was designed to estimate the baseline eGFR values of apparently health population living in the area with highest reported prevalence of CKDu in Sri Lanka using different estimation methods and also to compare the test characteristics of different methods of proteinuria/albuminuria estimations as valid field tests.

## Methods

Present paper included two components; secondary data analysis of routine screening programme in Anuradhapura and evaluation of proteinuria based screening methods used in combination with serum creatinine. This descriptive cross-sectional study was carried out as a part of population based screening for CKD in Anuradhapura in 2015. The screening was carried out as a part of the national programme on CKD prevention. Screening clinics were held in community settings easily accessible to the catchment population on pre-determined dates with prior notification given to the target population. Participation in the screening programme was voluntary and those who are without a know history of CKD were invited to participate. For the first component of this analysis, we selected only those who are above 18 years and having done both serum creatinine and urine SSA test to detect albuminuria as a part of screening programme. In 2015, SSA test was used on a spot urine sample routinely as a part of population based screening programme in this area. For serum creatinine, a 2 mL venous blood was drawn aseptically to a plain tube (with no anticoagulants) by a registered nursing officer. Serum samples were transported to the renal laboratory at teaching hospital, Anuradhapura same day and analyzed using Dimension RxL Pro clinical chemistry analyzer (Siemens Healthcare, Erlangen, Germany) by a trained medical laboratory technologist. SSA test was performed by adding 0.25 mL of urine to 1 mL of 30% sulfosalicylic acid and this method has been validated by the laboratory. Since study participants are not representative of age and sex structure of the population, we used direct standardization to estimate the prevalence values using the Sri Lankan population data.

The second component of this analysis was to assess the utility of urine based screening tests to use in community settings as stand alone screening tests, in comparison to serum creatinine based eGFR. Some of these tests were already in use as stand alone screening tests to detect CKD in Sri Lanka. All the relevant information including urine collection technique and urine containers were distributed among people by field health workers and a group of volunteers from each village, few days prior to the screening day. Informed written consent was obtained from all participants who participated in this additional procedure. All participants brought a morning urine sample (50 mL) using the containers provided and semi-quantitative measurement of urine protein was performed by using 2 para Uric-Techo (USA) urine strips and SSA test. Urine protein and albumin were measured by particle enhanced turbidimetric inhibition immunoassay technique and pyrogallol red method respectively on the same platform. Serum and urine creatinine assays were performed by the Jaffe method, which was traceable to isotope dilution mass spectrometry (IDMS) method, on Dimension RxL Pro clinical chemistry analyzer. Quality management was carried out appropriately including daily internal quality control runs and external quality assurance. A positive screening test was defined as having trace protein in USSA test or dipstick test, UACR > 30 mg albumin/g creatinine *or* UPCR > 150 mg protein/g creatinine.

eGFR was calculated using both MDRD (GFR (mL/min/1.73 m^2^) = 175 x (S_cr_/88.4)^-1.154^ x (Age)^-0.203^ x (0.742 if female) x (1.212 if African American) (SI units)) and CKD-EPI (GFR = 141 × min (S_cr_ /κ, 1)^α^ × max (S_cr_ /κ, 1)^-1.209^ × 0.993^Age^ × 1.018 [if female] × 1.159 [if black]) equations for comparison purposes. For description of eGFR in the population, we used Kidney Disease Improving Global Outcomes (KDIGO) guideline for classification of eGFR values [17]. All those who were having abnormal eGFR were repeated in three months and confirmed before the diagnosis was made. Comparison of two equations was done. To describe normality data of eGFR for the reference population, we presented age and sex disaggregated data and a linear regression model was fitted to describe the effect of age and sex on eGFR.

For the assessment of validity, first we consider eGFR as Gold Standard. However, eGFR alone is not 100% sensitive or specific to detect CKD. To overcome the inaccurate predictions of test characteristics due to imperfect Gold Standard tests, we used Bayesian latent class model (LCM) to estimates sensitivity, specificity and predictive values of the screening tests, including eGFR as well as screening tests as co-variates. The web based open access model developed by Modeling for Infectious disease Center (MICE) was used for this purpose (http://mice.tropmedres.ac/home.aspx). In this model, eGFR was also considered as imperfect test and model generated prevalence value (based on our inputs) were used for predictions of test validity.

Ethical clearance for the study was obtained from the Ethics Review committee of University of Peradeniya. All screen detected patients were referred to renal clinic and appropriate diagnostic tests were carried out.

## Results

The study sample included 7768 apparently healthy people aging 18 to 93 years and residing in Anuradhapura district. Females (*n* = 5522) accounted for 71.1% of the sample. Mean age of the participants was 45.9 (SD 14.1) years.

**Fig. 1:**
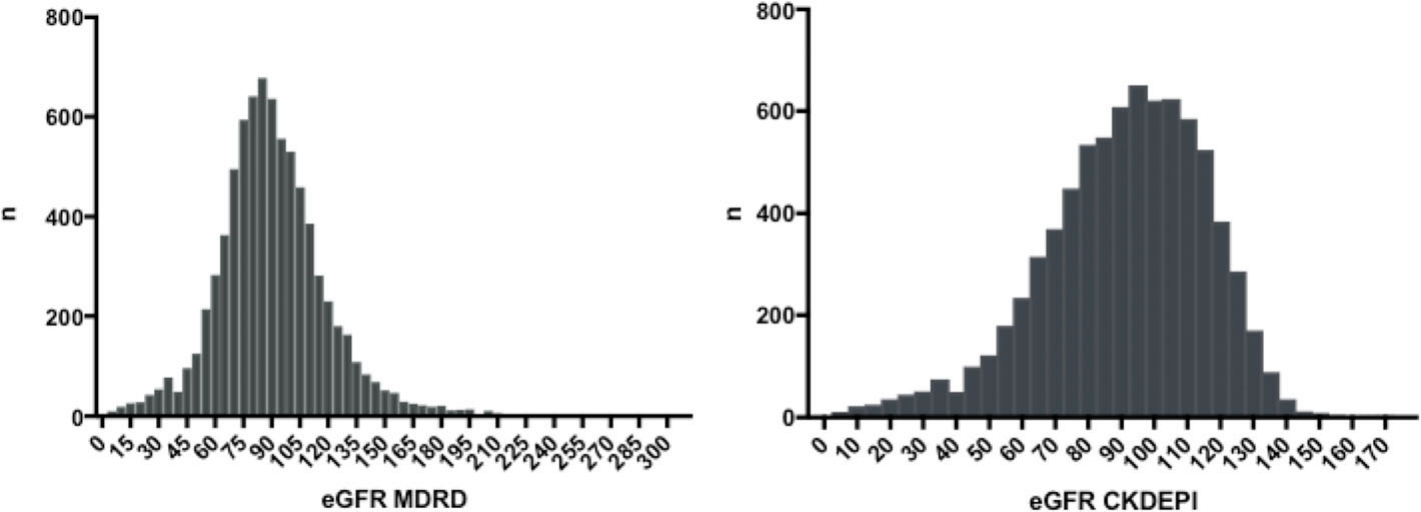
Distribution of eGFR using MDRD and CKD-EPI methods among 7768 screening participants from North Central Province, Sri Lanka

eGFR based on MDRD equation showed a normal distribution and a slightly skewed distribution was observed with CKD-EPI (Fig. 1).

**Fig. 2:**
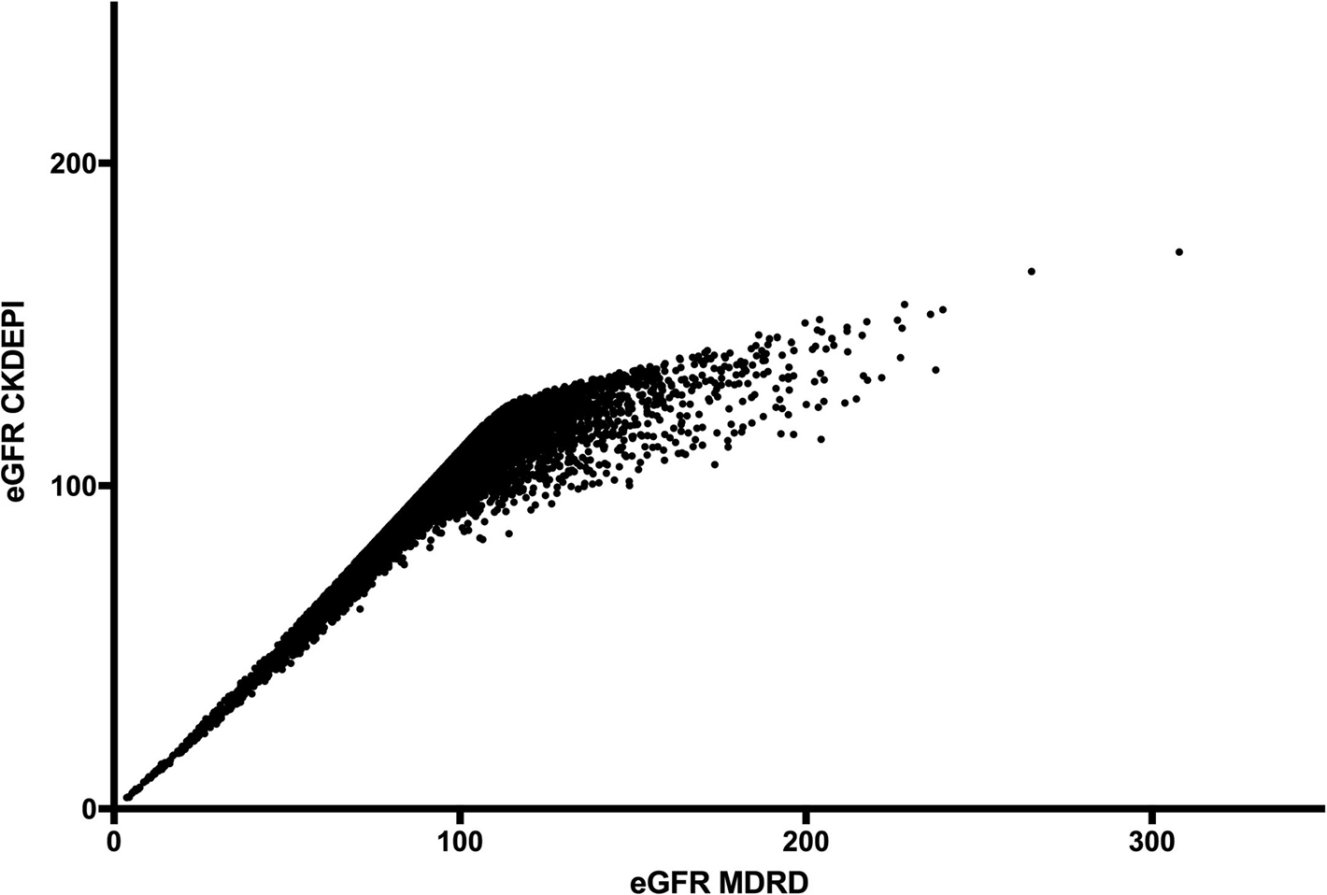
Comparison of eGFR values using MDRD and CKD-EPI formula among 7768 screening participants on North Central Province, Sri Lanka

Comparison of two equation showed that MDRD equation was progressively overestimating the values after eGFR of around 100 mL/min/1.73m^2^. The highest eGFR of 307 mL/min/1.73m^2^ in MDRD was reported as 172 mL/min/1.73m^2^ for the same patient using CKD-EPI equation (Fig. 2).

Subsequent analysis was done using eGFR calculated with CKD-EPI.

**Fig. 3:**
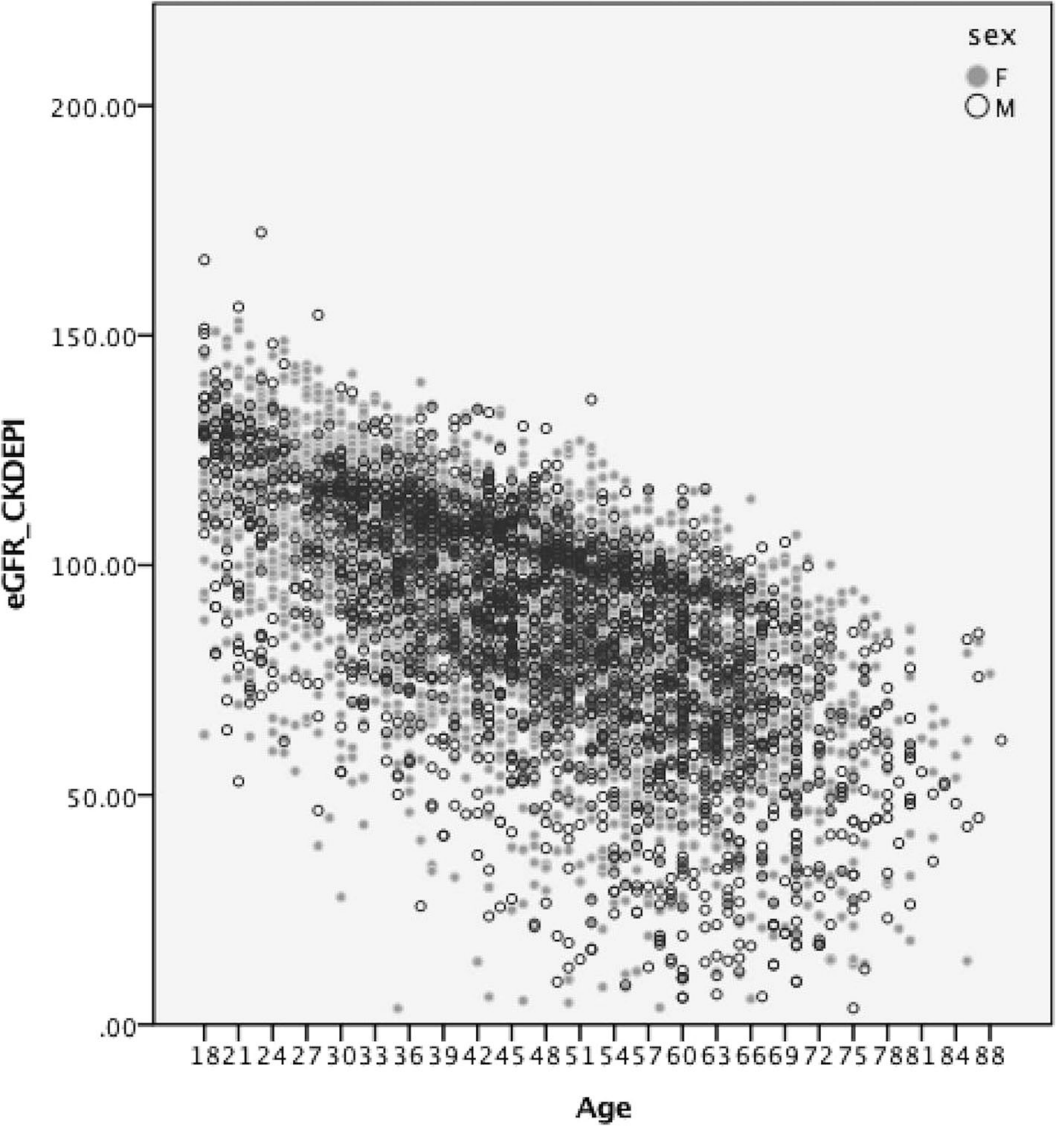
Distribution of eGFR by age and sex among 7768 screening participants on North Central Province, Sri Lanka

Age and sex disaggregated data showed a steady reduction of eGFR with wider data dispersion with increasing age in both sexes (Fig. 3).

Mean eGFR was 115 (SE .5) among people aging less than 30 and this value drastically reduced to 59.1 (SE 1.2) among people aging more than 70 years.

Males had a lower eGFR value in each age category compared to females. However, this observed difference was significant only in age categories 30–39, 50–59 and 60–69 years (Table 1).

**Table 1:** Age and sex disaggregated distribution of eGFR (CKD-EPI equation) values among 7768 apparently healthy CKD screening participants in Anuradhapura district

Age (years)	Sex		Mean	95.0% CL for Mean		Standard Deviation	Prevalence of eGFR<< 60 mL/min/1.73 m^2^ n %	
< 30	F	883	115.7	114.56	116.84	17.29	7	0.80
	M	291	112.79	110.52	115.06	19.66	4	1.40
30–39	F	1349	102.32	101.42	103.22	16.9	20	1.50
	M	447	99.43	97.76	101.11	18.04	15	3.40
40–49	F	1288	91.22	90.2	92.24	18.61	69	5.40
	M	541	89.57	87.82	91.32	20.73	49	9.10
50–59	F	1236	81.13	80.03	82.22	19.67	167	13.50
	M	505	76.18	74.13	78.23	23.39	103	20.40
60–69	F	597	73.57	72	75.14	19.48	133	22.30
	M	348	65.73	63.25	68.2	23.48	120	34.50
> = 70	F	169	61.88	58.6	65.16	21.6	68	40.20
	M	114	55.21	51.55	58.87	19.7	66	72.8

### Distribution of eGFR categories in the study sample

eGFR values were categorized according to the GFR categories for CKD staging according to KDIGO guidelines (Table 2). eGFR values compatible with moderate to severe decreased, severe decreased and kidney failure were detected among .7, 1.5 and 8.4% of the participants, totaling 10.6% prevalence of participants with eGFR value less than or equal to CKD Stage III.

**Table 2:** Distribution of eGFR categories among 7768 apparently healthy CKD screening participants in Anuradhapura district

eGFR level (mL/min/1.73m^2^)	n	%	95% confidence limits for proportion
G1 ≥ 90	4314	55.5	54.6- 56.8
G2 60–89	2633	33.9	32.7- 34.8
G3a 45–59	468	6.0	5.5- 6.6
G3b 30–44	188	2.4	2.1- 2.8
G4 15–29	113	1.5	1.2- 1.7
G5 ≤ 15	52	0.7	0.5- 0.9

Since the sample is over representing females and not representing the population structure, direct standardization was done to calculate the prevalence. For this purpose, we restricted the study population to 20–75 years age (*n* = 7450), because the number of study participants below and over that age group was too small for stable age specific rates. Prevalence of CKD in this restricted population was 10.3% and the age and sex adjusted (using Sri Lankan population structure) prevalence of eGFR less than 60 mL/min/1.73 m2 in this population was 10.6% (95% CI 9.9–11.3%). Estimated prevalence values for male and female populations were 12.2 and 9.2%.

Mildly decrease eGFR values were noted among 2633 (33.8, 95% CI 32.7–34.8%) of the participants. Among the latter group, urine protein (SSA method) was detected among 548 (21.4%). Progressively increasing proportions of proteinuria was detected among different categories (Table 3).

**Table 3:** Distribution of eGFR categories among 7768 apparently healthy CKD screening participants in Anuradhapura district by presence of proteinuria

eGFR level (mL/min/1.73m^2^)	Urine protein positive		Urine protein negative	
	*n*	%	*n*	%
Normal (> 90)	746	18.2	3359	81.8
Mildly decreased (60–89.9)	548	21.4	2007	78.6
Moderately to severe decrease (30–59.9)	240	37.6	399	62.4
Severely decreased (15–29.9)	85	75.9	27	24.1
Kidney failure < 15	40	78.4	11	21.6

### Evaluation of urine protein based test as screening test to detect patients with abnormal eGFR

For the evaluation of urine protein based test as screening tests, those who were having eGFR> 90 mL/min/1.73m^2^ were considered as normal and those who are having eGFR< 60 mL/min/1.73m^2^ were considered abnormal. We excluded all those people who were having eGFR 60–90 mL/min/1.73m^2^ from this analysis. This was done because it will create a bias result towards screening tests, since the urine protein is an added criteria for the diagnosis of CKD those who are having eGFR in the specified range From the second component of study, the Total number of patients available for this component was 730 with 126 patients with eGFr< 60 mL/min/1.73m^2^ Of the 730 selected patients, 498 (68.2%) were females. Age range was 18–85 years with a median age of 40.6 years (SD 15.5). Performance of individual tests are shown in Table 4.

**Table 4:** Performance of screening tests, in comparison to eGFR among CKD screening participants from Anuradhapura, Sri Lanka

	eGFR< 60 mL/min/1.73m^2^		eGFR> 90 mL/min/1.73m^2^	
	*n*	%	*n*	%
UACR				
> 30	45	35.7	40	6.6
= < 30	81	64.3	564	93.4
UPCR				
> 150	75	59.5	137	22.7
= < 150	51	40.5	467	77.3
Urine salicylic acid				
Trace or above	62	49.2	120	19.9
Negative	64	50.8	484	80.1
Urine dip stick^a^				
Trace or above	25	25.5	25	6.3
Negative	73	74.5	373	93.7

*eGFR* estimated glomerular filtration rate, *CKD* Chronic kidney disease, *UACR* Urine albumin creatinine ratio, *UPCR* Urine protein creatinine ratio. ^a^for 230 patients, urine dip stick results were not available

Sensitivity of UACR, UPCR, USSA and dip stick test to detect people with eGFR< 60 mL/min/1.73m^2^ was 35.7, 59.559.2 and 25.5% respectively. Since urine dipstick method was having very low sensitivity, we further evaluated other three screening tests using the Bayesian Latent Class model (Table 5).

**Table 5:** Test characteristics of UPCR, USSA and UACR as screening tests to detect renal impairment; Bayesian Latent Class model analysis

Test characteristic	UPCR	USSA	UACR
Sensitivity	61.8 (54.2–68.8)	50.9 (43.6–58.1)	35.3 (28.1–43.1)
Specificity	86.6 (82.5–90.9)	87.5 (83.7–91.1)	99.8 (98.3–100)
PPV	68.8 (58.3–79.6)	66.1 (55.6–76.2)	98.7 (90.2–100)
NPV	82.6 (77.1–86.9)	78.8 (73.1–83.5)	76.3 (70.0–81.5)

The test characteristics based on the Bayesian Latent Class model analysis shows that UPCR> 150 has the highest sensitivity. UACR, the usual recommended test as a screening test was having a sensitivity of 35.3% in this population.

## Discussion

In this study, we reported the normality data on eGFR in North Central Province of Sri Lanka, to be used as baseline data for future research and policy decisions. Further, the comparison of MDRD and CKD-EPI formula for eGFR estimation showed that the latter might be more applicable in screening “normal” population. The age and sex standardized prevalence of eGFR less than 60 mL/min/1.73 m2 in this population of adults aging more than 18 years was 10.6%. Finally, Bayesian model estimate shows that UPCR> 150 as the most suitable test to detect renal damage in this endemic settings.

Interpretation of our results should be done with the limitations inherited in the design we used. First, the objective of the screening programme was not prevalence estimates and the sample selected is not using a probability sampling techniques. So the prevalence estimate based on the present study sample may not be generalizable to the source population. Secondly, these estimates are about screening test positivity, not about confirmed CKD, which require repeated samples within three months. Those who are having GFR o60 ml/min/1.73 m^2^ may be due to CKD or acute kidney injury or both. Since these participants are apparently healthy, probability of having AKI is low. Further, we have not differentiated CKD and CKDu in this study due to unavailability of detailed clinical history. Lastly, the proper validation procedure includes comparison with “Gold Standard” test, which we have not done in this study. Rather, we compared the predictive probability of eGFR using urine based screening assays. The values reported should be interpreted as such.

Difference of MDRD and CKD-EPI formula in estimating eGFR is well known and studied. It is generally recommended for population as well as clinical use [18]. However, the differences in specific populations needs to be shown to get a good understanding about the population baseline data and interpretation. Our study shows the same pattern as previous studies done elsewhere. The eGFR estimation is based on age and sex, and the formula used has included these parameters to make sure that these changes are taken in to account. However, the population distribution data of eGFR by age and sex clearly shows that the cut off values for diagnosis of CKD may require adjustments. Using same threshold values for all age groups may lead to gross overestimation of CKD in this population, which might lead to issue in public health interventions. The National Kidney Foundation KDOQI guideline clearly states that age distribution of GFR values should be considered for threshold values for CKD diagnosis [19]. The need for age base threshold values for CKD is discussed in literature [20] but yet to be implemented in health systems.

Leakage of albumin is caused by the involvement of the glomeruli in the initial stages of the disease due to common causes such as diabetes and hypertension. Evidence exists to suggest that the initial disease mechanism in CKDu primarily does not involve the glomeruli [11]. It is caused by chronic tubulointerstitial nephritis where proteinuria or albuminuria is not common as with conventional causes of CKD in initial stages. Among this study population where CKDu is the predominant disease, lack of microalbuminuria or proteinuria is challenging for early diagnosis in community settings. This study clearly demonstrated that standard cut off value looking for microalbuminuria (UACR> 30) is less sensitive to detect early cases. Multiple test will yield more results, however UPCR is recommended in addition to UACR as a screening test in this population where CKDu is common, specially because the available evidence [21–23] clearly suggests that the mortality and morbidity predictions needs combination of GFR and albuminuria/proteinuria. Hence, the ideal should be to use both eGFR and UACR/UPCR in this population.

## Conclusions

We recommend further studies to investigate age and sex specific adjustments for screening and diagnostic cutoff values for eGFR. UPCR and UACR should be use as screening tests in areas with high proportion of CKDu patients to increase the yield of the screening programme. However, combination of eGFR and UPCR/UACR should be the ideal screening method.

## Data Availability

The datasets used and/or analyzed during the current study are available from the corresponding author on reasonable request.
